# Microconfined Assembly of High‐Resolution and Mechanically Robust EGaIn Liquid Metal Stretchable Electrodes for Wearable Electronic Systems

**DOI:** 10.1002/advs.202402818

**Published:** 2024-06-19

**Authors:** Jingxuan Ma, Zicheng Sa, He Zhang, Jiayun Feng, Jiayue Wen, Shang Wang, Yanhong Tian

**Affiliations:** ^1^ National Key Laboratory of Precision Welding & Joining of Materials and Structures Harbin Institute of Technology Harbin 150001 China; ^2^ Department of Mechanical Engineering The University of Hong Kong Hong Kong 999077 China; ^3^ Advanced Biomedical Instrumentation Centre Limited Hong Kong 999077 China; ^4^ Zhengzhou Research Institute Harbin Institute of Technology Zhengzhou 450041 China

**Keywords:** electrodeposition, electrohydrodynamic printing, flexible electronics, liquid metal, multilayer circuits

## Abstract

Stretchable electrodes based on liquid metals (LM) are widely used in human‐machine interfacing, wearable bioelectronics, and other emerging technologies. However, realizing the high‐precision patterning and mechanical stability remains challenging due to the poor wettability of LM. Herein, a method is reported to fabricate LM‐based multilayer solid–liquid electrodes (m‐SLE) utilizing electrohydrodynamic (EHD) printed confinement template. In these electrodes, LM self‐assembled onto these high‐resolution templates, assisted by selective wetting on the electrodeposited Cu layer. This study shows that a m‐SLE composed of PDMS/Ag/Cu/EGaIn exhibits line width of ≈20 µm, stretchability of ≈100%, mechanical stability ≈10 000 times (stretch/relaxation cycles), and recyclability. The multi‐layer structure of m‐SLE enables the adjustability of strain sensing, in which the strain‐sensitive Ag part can be used for non‐distributed detection in human health monitoring and the strain‐insensitive EGaIn part can be used as interconnects. In addition, this study demonstrates that near field communication (NFC) devices and multilayer displays integrated by m‐SLEs exhibit stable wireless signal transmission capability and stretchability, suggesting its applicability in creating highly‐integrated, large‐scale commercial, and recyclable wearable electronics.

## Graphical abstract

Liquid metal based multilayer solid–liquid electrodes (m‐SLE) are fabricated through electrohydrodynamic (EHD) printed confinement template, which is composed of PDMS/Ag/Cu/EGaIn. The high resolution and high stretchability of m‐SLE enable it to be manufactured into human health sensing, near field communication (NFC) and wearable flexible display devices.

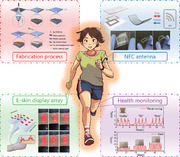

## 1 Introduction

Stretchable electrodes are essential for fabricating electronic products with high conformal adaptability at strain state, which are widely applied in human‐machine interfacing,^[^ ^1^, ^2^, ^3^, ^4^ ^]^ wearable bioelectronics,^[^ ^5^, ^6^, ^7^, ^8^, ^9^, ^10^ ^]^ soft actuators,^[^ ^11^, ^12^, ^13^ ^]^ and other advanced technologies.^[^ ^14^, ^15^, ^16^, ^17^ ^]^ Liquid metals (LM) display a set of remarkable properties, such as high ductility, high electrical conductivity, and biocompatibility,^[^ ^18^, ^19^, ^20^, ^21^ ^]^ and recent research has begun to explore its feasibility as conductive components in stretchable electrodes, creating new opportunities for wearable monitoring device,^[^ ^22^, ^23^, ^24^, ^25^, ^26^ ^]^ nanogenerators,^[^ ^27^, ^28^, ^29^, ^30^ ^]^ opto‐electronics^[^ ^31^, ^32^, ^33^, ^34^ ^]^ and energy‐storage devices.^[^ ^35^, ^36^, ^37^, ^38^, ^39^ ^]^ However, despite extensive research efforts, high‐precision patterning LM on stretchable substrates with a line width less than 20 µm remains challenging, which limits their practical applications, due to its fluidity nature and high surface tension.^[^ ^40^ ^]^ For example, LM features fabricated by conventional screen‐printing methods usually exhibit a resolution at the level of ≈200 µm,^[^ ^41^ ^]^ which is significantly lower than that of other nanoinks (e.g., silver^[^ ^42^ ^]^ or copper nanoparticles^[^ ^43^ ^]^). This discrepancy is due to the fact that the high surface tension makes it is difficult to push LM into the screen opening. The weak adhesive strength between LM pattern and substrate also makes them prone to detachment during long‐term usage, leading to mechanical stability issues.^[^ ^44^ ^]^ Moreover, the printed features usually required extra post treatments for improving the conductivity (e.g., thermal, ultrasonic sintering), which raised the economic and time cost.^[^ ^45^, ^46^, ^47^ ^]^

Recently, selective metallization on copper templates was exploited for realizing highly conductive and mechanically robust LM features.^[^ ^48^, ^49^, ^50^ ^]^ For example, patterning LM onto super‐hydrophilic laser‐induced graphene film with copper plating led to a highly conductive and stretchable LM circuit with a sheet resistance of 3.54 mΩ per square without additional sintering process.^[^ ^51^ ^]^ In another example, reactive wetting Galinstan LM onto silver/copper hybrid template resulted in the fabrication of LM features with remarkable electrical conductivity and mechanical durability.^[^ ^52^ ^]^ However, these methods still exhibited low resolution (e.g., ≈0.1 mm) significantly due to the large line width of the template. It was still difficult to realize highly conductive and mechanically robust LM patterns onto stretchable substrate without redundant post treatments.

Here, we present a different fabricating process for creating high‐resolution LM circuit with desired electrical conductivity and mechanical durability for device manufacturing. In this scheme, advanced electrohydrodynamic (EHD) printing was utilized for guiding the confined patterning of LM, resulting in an ultrathin linewidth of 19.28 µm. The obtained multilayer solid–liquid electrodes (m‐SLE) demonstrated remarkable strain insensitivity up to 100% strain, and maintained almost constant resistance throughout 10 000 tensile/relaxation cycles. Simultaneously, the multi‐layered structure of m‐SLE enabled the adjustable strain sensing capabilities. The strain‐sensitive Ag component could be utilized as a sensor in human health monitoring, while the strain‐insensitive EGaIn component served as interconnects to shield non‐detective areas from external disturbances. Furthermore, the simple fabrication process of these m‐SLE allow the board applicability in flexible devices, including human physiological sensors, wireless power supply NFC antenna, and a wearable LED display array. The remarkable electronic and mechanical performance of these m‐SLE, in conjunction with their manufacturability and recyclability provides new potentials for the future development and processing of high‐performance, highly reliable, multilayer, multifunctional and recyclable commercial applications.

## 2 Results and Discussion

### 2.1 Fabrication and Structures

**Figure 1:**
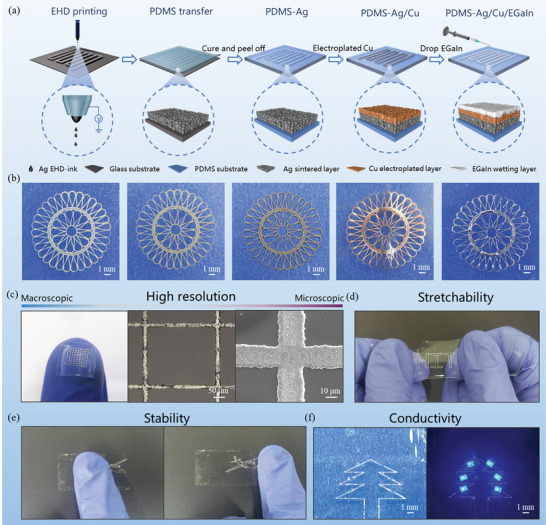
Fabrication and structures of m‐SLE. a) Schematic illustration of fabrication process involving EHD printing, transfer, electrodeposition, and EGaIn selectively wetting. b) Corresponded optical images at each step. Demonstration of m‐SLE with c) high resolution, d) stretchability, e) adhesion ability and f) conductivity.

The fabrication process of m‐SLE is schematically illustrated in **Figure** **1**a. Fabrication of the high‐precision m‐SLE exploit Ag/Cu template with a precision less than 20 µm involving EHD printing of silver nano ink and electrodeposition of copper layer. EHD printing of silver nanoparticles (AgNPs) (Figure S1, Supporting Information) followed by transferring onto PDMS substrate enables the fabrication of stretchable Ag electrodes (Figure S2, Supporting Information). Electrodeposition of Cu layer can improve the wettability of EGaIn and enhance the interfacial adhesion. Under an appropriate current density (0.15 mA cm^−2^), the Cu layer was dense and completely covered the bottom Ag. The coating layer resolution was basically the same as before electrodeposition. Detailed characterization of the Cu electrodeposition process is shown in Figure S3 (Supporting Information). Next, EGaIn was facilely casted onto the surface of obtained PDMS/Ag/Cu trilayer template. Interestingly, EGaIn selectively wetted at the Ag/Cu patterned region and forming ultrafine features. The detailed process of selective wetting of liquid metal on the Cu surface is shown in Figure S4 and Video S2 (Supporting Information). Successful confined fabrication of high‐precision m‐SLE was confirmed by the corresponded optical images of exquisite carved pattern exhibited in Figure 1b. The deposition of each layer can be easily distinguished by visual color changes. As exhibited by the scanning electron microscopy (SEM) in Figure 1c, the edge and corner of the pattern were sharp and distinct. The robustness of m‐SLEs was also indicated into the macroscopic performance, as exemplified by excellent stretchability (Figure 1d), adhesion ability (Figure 1e) and conductivity (Figure 1f).

**Figure 2:**
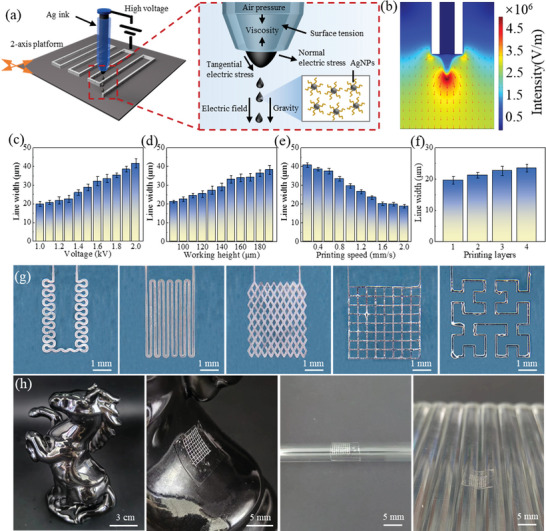
EHD printing of Ag pattern and representative m‐SLE patterns. a) Schematic diagram of EHD printing mechanism of Taylor cone generation. b) Finite element simulation of electric field intensity during Taylor cone generation. Parameter optimization of EHD printing, including c) voltages, d) working heights, e) printing speeds and f) printing layers. g) Representative m‐SLE patterns prepared with optimized EHD printing parameters, including horseshoe curves, serpentine curves, rhombic grids, square grids and third‐order Hilbert curves. h) Grid patterns on horseback, a glass tube and inclined surfaces.

The key for fabricating m‐SLEs is the EHD printing process of Ag patterns, which provide the confined region for subsequent high‐resolution patterning Cu and EGaIn. As shown in **Figure** **2**a, the electrostatic field between the nozzle and substrate exerts a downward electric field force on the conductive Ag nanoink, allowing it to overcome the effects of viscous force and surface tension, forming a Taylor cone that is ejected onto the substrate surface. Finite element simulation was employed to analyse the electric field distribution during the EHD process, confirming that the strongest electric field is present at the tip of the Taylor cone. This facilitates a continuous ejection of ink, leading to continuous printing. To achieve high‐resolution silver patterns, we conducted a thorough analysis of the impact of voltage, working height, printing speed, and number of printing layers. It was found that increasing the printing speed and reducing voltage, working height, and number of printing layers contribute to reducing line width (Figure 2c–f). As exhibited in Figure 2g, various high‐precision and exquisite m‐SLE patterns, including horseshoe curves, serpentine curves, rhombic grids, square grids, and third‐order Hilbert curves, are achieved. Furthermore, the soft substrate could conform to complex curved surface, as exemplified by the tight attachment onto horseback, glass tube, inclined surfaces (Figure 2h), glass hemisphere, the corner of a glass bent tube and a corrugated surface (Figure S5, Supporting Information). The m‐SLE patterns exhibit a high electrical conductivity of 3.1 × 10^4^ S cm^−1^. The final printed dimensions and density are adjustable. Figure S6 (Supporting Information) shows the morphology and conductivity of m‐SLE based grid electrodes with different printing line widths (20, 40, 60, 80, 100 µm) and pitches (300, 400, 500, 600, 700 µm). In addition to EGaIn, the m‐SLE manufacturing process proposed in this study is also applicable to other liquid metals, such as eutectic gallium tin (EGaSn) and gallium indium tin alloy (Galinstan). The SEM and surface scan results of m‐SLE based on EGaIn, EGaSn, and Galinstan are shown in Figure S7 (Supporting Information).

**Figure 3:**
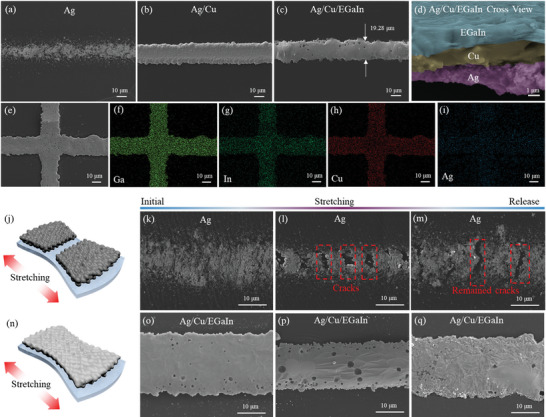
Microstructures of m‐SLE. SEM images of m‐SLE fabrication at different stages, namely a) PDMS‐Ag, b) PDMS‐Ag/Cu and c) PDMS‐Ag/Cu/EGaIn (m‐SLEs). d) Cross‐sectional SEM image of m‐SLE. e–i) EDS mapping of m‐SLE. j) Schematic diagram of PDMS‐Ag in stretching state. SEM images of PDMS‐Ag in k) initial, l) stretching and m) release state. n) Schematic diagram of m‐SLE in stretching state. SEM images of m‐SLE in o) initial, p) stretching and q) release state.

SEM analysis (**Figure** **3**a–c) confirmed the sequential deposition of Cu and EGaIn layers onto the high‐resolution Ag pattern. Some minor cracks and irregular edges were observed in the pristine Ag line, which can be attributed to the expansion of the PDMS prepolymer during the thermal curing process. The electrodeposition encapsulation of Cu layer onto Ag pattern serves as a selective wetting template for EGaIn and exhibited a neat and consistent pattern morphology. Figure 3c illustrates the precise deposition of the EGaIn pattern on the Ag/Cu template with an ultrathin width of 19.28 µm, confirming its self‐assembling property to form high‐resolution multilayer conductive circuits. The corresponded optical microscopy (OM) images can be observed in Figure S8 (Supporting Information). The cross‐sectional view in Figure 3d and Figure S9 (Supporting Information) reveals the multilayer structure of m‐SLEs, comprising EGaIn, Cu, and Ag layers, with approximate thicknesses of ≈2 µm. Uniform elemental distribution including Ga, In, Cu, and Ag at intersection region also confirmed the successful fabrication of m‐SLEs. Subsequently, we characterized the microstructures of PDMS‐Ag (without Cu and EGaIn treatment) and m‐SLE. It was found that PDMS‐Ag exhibited irreversible crack formation during stretching, which persisted even after release. In contrast, m‐SLE showed no observable crack formation during stretching, which can be attributed to the superior flowability of the upper EGaIn layer.

### 2.2 Electromechanical Properties

**Figure 4:**
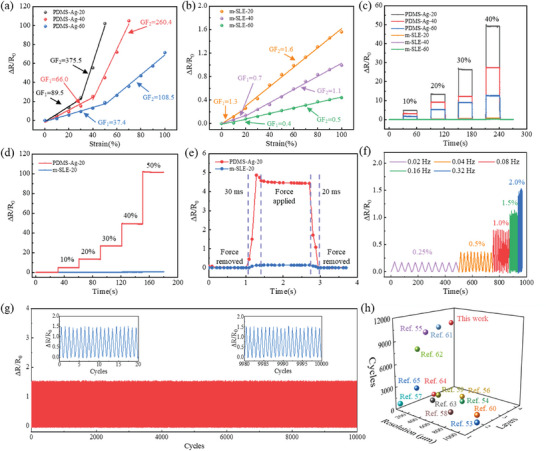
Sensing properties of PDMS‐Ag and m‐SLE based strain sensor. a) Resistive response of PDMS‐Ag with different line widths (20, 40, 60 µm). b) Resistive response of m‐SLE with different line widths (20, 40, 60 µm). c) Resistive response of PDMS‐Ag and m‐SLE strain sensors with different line widths under cyclic load strains of 10%, 20%, 30%, and 40% respectively. d) Resistive response of PDMS‐Ag‐20 and m‐SLE‐20 strain sensors under a series of stepwise increasing strains from 10% to 50%. e) Resistive response of PDMS‐Ag‐20 and m‐SLE‐20 strain sensors when applying and releasing 10% strain. f) Resistive response of PDMS‐Ag strain sensor at different frequencies (0.02. 0.04, 0.08, 0.16, 0.32 Hz) and strains (0.25%, 0.5%, 1.0%, 1.5%, 2.0%). g) Cycling stability of m‐SLE sensor at 100% strain within 10 000 cycles. The insets show the first and last 20 cycles respectively. h) Comparison of the resolution, number of functional layers, and cycling stability with reported literature.^[^ ^53^, ^54^, ^55^, ^56^, ^57^, ^58^, ^59^, ^60^, ^61^, ^62^, ^63^, ^64^, ^65^ ^]^

We investigated the relative resistance change (ΔR/R_0_) of PDMS‐Ag and m‐SLEs during stretching. Specifically, a series of serpentine patterns with different line widths (20, 40, and 60 µm) were prepared by controlling EHD printing parameters, namely PDMS‐Ag‐20, PDMS‐Ag‐40, PDMS‐Ag‐60, and m‐SLEs‐20, m‐SLEs‐40, m‐SLEs‐60, respectively (Figure S10, Supporting Information). For both PDMS‐Ag and m‐SLE electrodes, the ΔR/R_0_ value exhibits an upward trend as the line width decreases (**Figure** **4**a,b). The sensitivity of the sensor is defined as the gauge factor (GF), GF = (ΔR/R_0_)/ε × 100%, where ε represents the strain magnitude. By fitting the data from Figure 4a,b in the manuscript, the sensitivity under different strain ranges was obtained. For PDMS‐Ag‐20, the GF was 89.5 in the strain range of 0–30%, and 375.5 in the strain range of 30–50%. For m‐SLE‐20, the sensitivity was 1.3 in the strain range of 0–20%, and 1.6 in the strain range of 20–100%. With increasing line width, the resistance response value decreased at the same strain. However, the PDMS‐Ag structure had a limited strain sensing range due to its susceptibility to irreversible damage under excessive strain. The maximum strain sensing ranges for PDMS‐Ag 20 and PDMS‐Ag 40 were 50% and 70%, respectively. In contrast, m‐SLE exhibited much smaller changes in resistance under the same strain and has a larger strain sensing range. m‐SLE‐20, m‐SLE‐40, and m‐SLE‐60 could all detect up to 100% strain. Moreover, we assessed the dynamic sensing stability and reliability of both PDMS‐Ag and m‐SLE under cyclic tensile/relaxation loads at varying strain levels of 10%, 20%, 30%, and 40% (Figure 4c). PDMS‐Ag demonstrated consistent response to specific strains within the 0–40% strain range, indicating its potential for accurately detecting specific motion or gesture applications. In contrast, m‐SLE exhibited a higher insensitivity to strain variations, suggesting its potential for signal transmission without being affected by strain interference. The resistance changes were tested for PDMS‐Ag‐20 and m‐SLEs‐20 under gradually applied strains (Figure 4d). PDMS‐Ag exhibited excellent sensing response characteristics, while m‐SLEs displayed good electrical stability. It is worth noting that both PDMS‐Ag and m‐SLEs exhibited shorter response times (30 ms) and recovery times (20 ms) at 10% strain (Figure 4e). The ΔR/R_0_ variation showed strict consistency at each strain (0.25%, 0.5%, 1.0%, 1.5%, 2.0%) and at each frequency (0.02, 0.04, 0.08, 0.16, 0.32 Hz), indicating the excellent stability of m‐SLEs at different cyclic frequency (Figure 4f). The strain detection of PDMS‐Ag‐20 is very accurate, detecting micro‐strain down to 0.25%. After 10 000 tensile/relaxation test cycles at 100% strain, the measurement signal of m‐SLE showed no significant degradation in the shape and peak of the electrical response (Figure 4g; Figure S11, Supporting Information), demonstrating excellent dynamic stability and sensing performance. as wearable strain sensors. The cyclic stability of PDMS‐Ag was also tested, as shown in Figure S12 (Supporting Information). PDMS‐Ag has good cycle stability under 30% strain, and the signal output remains highly repeatable after 10 000 cycles. Moreover, PDMS‐Ag and m‐SLE were also confirmed to have long‐term storage reliability (Figure S13, Supporting Information). Furthermore, a comparison of our work with other reported patterned liquid metal sensor^[^ ^53^, ^54^, ^55^, ^56^, ^57^, ^58^, ^59^, ^60^, ^61^, ^62^, ^63^, ^64^, ^65^ ^]^ demonstrates its superior overall performance (Figure 4h; Table S1, Supporting Information). While some methods achieve superior resolution,^[^ ^64^, ^65^ ^]^ our approach offers optimal performance when considering sensor capabilities, versatility in applications, and sustainability through reusability.

### 2.3 Applications as Wearable Sensors

**Figure 5:**
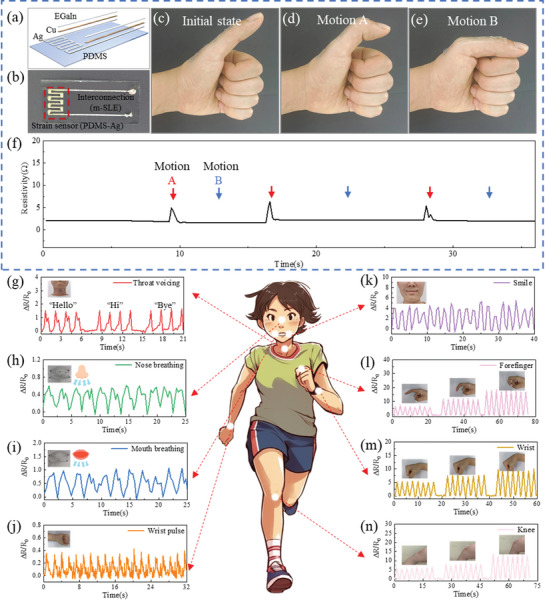
The wearable m‐SLE strain sensors for human health monitoring. a) Schematic illustration of the composition of the finger joint strain sensor. b) Optical image of the finger joint strain sensor. Optical images showing c) the initial state, d) bending of the first joint (Motion A) and e) bending of the second joint (Motion B) of thumb. f) Resistance changes of a strain sensor mounted on the thumb during cyclic bending of the first and second joints. The red arrow (Motion A) and the blue arrow (Motion B) represent the time points of bending of the first joint and the second joint respectively. Resistance changes during health monitoring in different parts of the human body, namely g) throat, h) nose breathing, i) mouth breathing, j) pulse, k) smile, l) index finger bending, m) wrist bending and n) knee bending.

Electronic devices designed to adhere to skin typically require two essential components: strain‐sensitive structures for sensing and strain‐insensitive structures for stretchable electrical interconnections. This design ensures undisturbed electrical signals even during repetitive deformation of the skin. After conducting electromechanical performance tests, it was determined that PDMS‐Ag is well‐suited as the strain‐sensitive component for the sensing part, while m‐SLE is suitable for the interconnections due to its ability to minimize resistive fluctuations in response to external mechanical stress, thereby optimizing the signal‐to‐noise ratio. To demonstrate this concept, a skin sensor device was fabricated, incorporating a strain‐sensitive element made of PDMS‐Ag and a stretchable interconnection made of m‐SLE (as shown in **Figure** **5**a,b). This device accurately recorded induced strain at a specific position on the thumb. It was attached to the thumb, spanning two joints, with the strain sensor placed at the proximal joint and the interconnection at the distal joint (Figure 5c; Figure S14, Supporting Information). When the first joint bent (Motion A), the device detected a change in resistance due to the deformation of the strain sensor (Figure 5d,h). However, when the second joint bent (Motion B), causing stretching of the interconnect, no significant variation in resistance was observed (Figure 5e,h). More details are presented in Video S3 (Supporting Information).

Based on the aforementioned integrated strain sensor with PDMS‐Ag and m‐SLE, we applied it to volunteers to assess its capabilities for human health monitoring. The strain sensors were attached to the volunteers throats and asked them to articulate “Hello,” “Hi,” and “Bye,” four times each. The resulting data exhibited consistent signal characteristics across all iterations, indicating the sensor's capability for reliable voice recognition (Figure 5g). Additionally, the sensors demonstrated their ability to capture stable electrical signals corresponding to subtle human movements, such as nose breathing, mouth breathing, wrist pulse, and smiling (Figure 5h–k). Significant responses were observed for these weak signal peaks, indicating the potential of the sensor for assessing medical health monitoring applications. We further evaluated the sensor's performance through bending tests involving the index finger, wrist, and knee. The corresponding dynamic responses are shown in Figure 5l–n. In these movements, all responses gradually increased with increasing bending angles. Moreover, the bending tests demonstrated the high flexibility of the sensor, demonstrating its potential for biocompatible applications.

### 2.4 Applications as NFC Devices and Stretchable Multilayer Display

**Figure 6:**
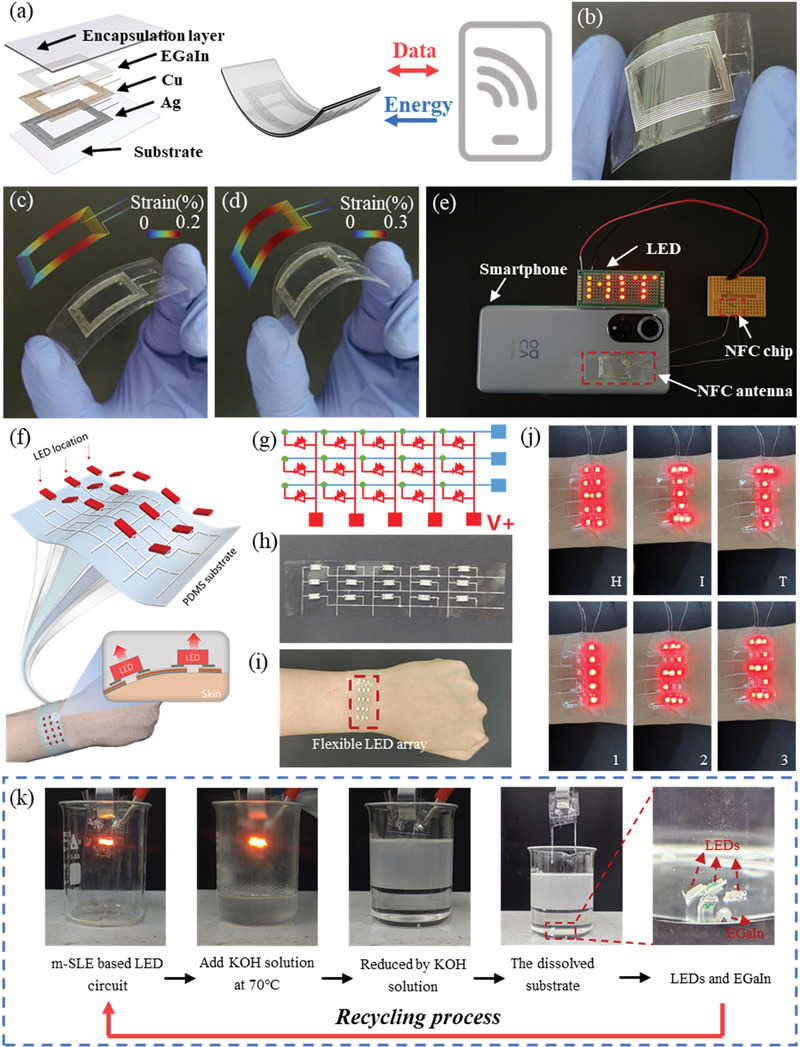
Application of m‐SLE in NFC antenna and E‐skin display array and its recycling strategy. a) Fabrication of an m‐SLE‐based NFC antenna, which enables wireless interaction with a smartphone, harnessing its energy to light up LEDs. b) Photograph of m‐SLE‐based NFC antenna. Optical images of NFC tags under c) small and d) large bends and the corresponding strain distribution under bending (inset). e) The photograph that shows the power transmitted from an NFC‐enabled smartphone to an m‐SLE‐based NFC antenna can light up 19 parallel‐connected LEDs. f) Schematic illustration of m‐SLE‐based E‐skin display array. g) Schematic layout of the m‐SLE‐based E‐skin display array. h) Optical image of the m‐SLE‐based E‐skin display array. i) E‐skin display array applying to human wrist. j) The display shows the letters “H”, “I”, “T” and the numbers “1”, “2”, “3”. k) Recycling strategy of EGaIn in m‐SLE‐based flexible devices.

In the era of the Internet of Things (IoT), integrating of high‐performance antennas is crucial for enhancing the functionality of flexible, portable electronic devices. These antennas enable wireless data transfer and power collection. Near‐field communication (NFC) enables the simultaneous transference of power and information among devices via inductive coupling, thus establishing a platform for battery‐independent microsensor electronics. To meet this demand, we fabricated an NFC antenna using m‐SLEs with a width of 18 mm and a length of 27 mm (**Figure** **6**a,b). We chose specific geometric parameters, including eight coil turns, a line width of 50 µm, and a coil spacing of 0.5 mm (Figure S15, Supporting Information). Finite element simulations demonstrated that strain primarily occurred along the axial direction during deformation (Figure 6c,d). Importantly, the antenna remained intact without any cracks during the bending process (Figure S16, Supporting Information). Our m‐SLEs‐based NFC antenna successfully established stable communication with various NFC‐enabled devices. Notably, it showed excellent energy transmission capabilities by efficiently harvesting energy from a smartphone and lighting up a pattern composed of 19 LEDs, displaying the word “HIT” (Figure 6e; Video S4, Supporting Information). These findings highlight the advantages of our m‐SLEs NFC antenna over commercial alternatives and its potential in battery‐independent wireless sensors.

Furthermore, we presented a highly integrated multi‐layer E‐skin display screen composed of m‐SLE and a matrix LED array (Figure 6f). This device consisted of three rows of m‐SLEs lines (X) and five columns of m‐SLEs lines (Y), connected to either end of the LEDs, forming a 3×5 cross‐grid display array with 15 pixels. We demonstrated its application by attaching the multi‐layer E‐skin display array to a volunteer's wrist, with the ability to digitally render letters “H,” “I,” “T,” and numbers “1,” “2,” “3” (Figure 6j; Video S5, Supporting Information).

The increasing demand for disposable wearable devices, which are typically designed for single‐use to mitigate potential infection risks associated with repeated use, presents substantial economic and environmental challenges in waste management. To address this issue, zero‐waste medical electronics made from recyclable materials have received widespread attention. LM has emerged as a promising material in this domain, capable of being recycled into bulk LM through acid or alkali treatment. Based on the high‐precision patterning of m‐SLE, we demonstrated its potential in functionalizing soft zero‐waste wearable electronic products. Figure 6k illustrates the EGaIn recycling strategy within m‐SLE. The flexible circuit consisted of four LEDs, with the m‐SLE structure serving as the interconnect. The results revealed that after immersing the circuit in a KOH solution (70 °C), the PDMS substrate dissolved, the m‐SLE fractured, and the LEDs turned off. Eventually, the portion immersed in the KOH solution completely dissolved. EGaIn agglomerated into spherical forms while retaining their original fluidity and conductivity. The recycled EGaIn can be reused multiple times.

## 3 Conclusion

In summary, we have demonstrated high‐precision patterning LM assisted by EHD printed confinement template for the construction of stretchable m‐SLEs. The obtained electrodes combine high resolution, excellent mechanical stability, and recyclability for precise, reliable, recyclable device manufacturing. The versatile m‐SLEs wit multilayer structure are precisely aligned among layers from top to down, which may expand the toolbox for the development of multilayer stretchable electrodes. Furthermore, the high‐precision multi‐layer m‐SLE possessed adjustable strain sensitivity. The initially prepared Ag lines, being sensitive to strain, were suitable for physiological monitoring. The subsequently formed EGaIn layer, insensitive to strain, served as interconnects to shield disturbances from outside the detection area. We also demonstrated that the near‐field communication (NFC) devices and multi‐layer displays integrated by m‐SLE exhibited stable wireless signal transmission capabilities and stretchability, indicating their suitability for creating highly integrated, large‐scale commercial, and recyclable wearable electronic products. It is worth noting that fabrication process does not involves toxic methods and chemicals as those needed in other stretchable electrodes, which makes m‐SLEs favourable for further application in vivo bioelectronics, such as tissue engineering technology. Further tuning the substrate types, line width, mesoscale patterning, or other attributes of m‐SLEs may open new opportunities for emerging flexible electronics.

## 4 Experimental Section

### Materials

All employed chemical reagents were utilized in their as‐received state, without any additional purification processes. Silver nitrate (AgNO_3_), ferrous sulfate (FeSO_4_), trisodium citrate (C_6_H_5_Na_3_O_7_) and ethanol were obtained by Sinopharm Chemical Reagent Co., Ltd. (Beijing, China). Ethylene glycol (EG) was provided by Tianjin Fengchuan Chemical Reagent Technology Co., Ltd. (Tianjin, China). Gallium (Ga, 99.99%), Indium (In, 99.99%) and Tin (Sn, 99.99%) were obtained from Shanghai Macklin Biochemical Co., Ltd. EGaIn was prepared by melting a mixture of Ga and In with a weight ratio of 75.5:24.5 at 120 °C for 2 h. EGaSn was prepared by melting a mixture of Ga and Sn with a weight ratio of 91.2:8.8 at 120 °C for 2 h. Galinstan was prepared by melting a mixture of Ga, In, and Sn in a weight ratio of 68.5:21.5:10 at 120 °C for 2 h. PDMS was obtained from Dow Corning Co., Ltd. (Midland, America).

### The Preparation of AgNPs and AgNP‐Based Conductive Ink

AgNPs was synthesized using a chemical reduction method, employing AgNO_3_ as the source of silver, FeSO_4_ as the reducing agent, C_6_H_5_Na_3_O_7_ as the dispersing agent, and water as the solvent. The synthesis proceeded as follows: Initially, solutions of 30 mL of 0.5 mol L^−1^ AgNO_3_, 30 mL of 0.5 mol L^−1^ FeSO_4_, and 30 mL of 1 mol L^−1^ C_6_H_5_Na_3_O_7_ were prepared. Subsequently, the solutions of AgNO_3_ and C_6_H_5_Na_3_O_7_ were combined and subjected to vigorous stirring. The FeSO_4_ solution was gradually added to the mixture, and the reaction was allowed to proceed for 30 min. Following this, the AgNPs were isolated through multiple rounds of centrifugal washing. Ultimately, the AgNPs were redispersed in a solvent mixture of EG and deionized water (EG:deionized water = 7:3).

### The Preparation of Ag Conductive Patterns by EHD Printing

The fabrication of Ag conductive patterns was carried out utilizing an EHD platform (HEIJ‐H, Wuhan Huaweike Intelligent Technology Co., Ltd.). AgNP‐based conductive ink was filled in a metal nozzle (with diameter of 60 µm). And the metal nozzle was connected to an air pump and a voltage generator. The initiation of the printing process required the application of a high voltage across the nozzle and the substrate. After the EHD printing process, post‐processing of the Ag pattern was performed at 175 °C for 30 min.

### Fabrication of m‐SLE

The preparation process of m‐SLE consisted of four sequential steps. First, the prepared Ag ink was EHD‐printed on the surface of a glass substrate. The Ag pattern was achieved through the adjustment of voltage, printing height, and printing speed. Second, 2 mL of PDMS prepolymer (10:1) was spin‐coated on the top of the printed pattern (1 krpm 60 s) and cured at 80 °C oven for 2 h. Then, it was detached from the glass substrate, resulting in a PDMS/Ag bilayer composite structure. Subsequently, the PDMS/Ag structure was immersed in a Cu electrodeposition solution, and Cu was electrodeposited under constant current density. At this stage, the PDMS/Ag/Cu structure was attained. Lastly, EGaIn was uniformly applied to the surface of the PDMS, wherein EGaIn selectively wetted the Cu surface while refraining from wetting the remaining PDMS surface. Consequently, the PDMS/Ag/Cu/EGaIn structure, known as m‐SLE, was obtained.

### Materials Characterization

The top view and cross‐sectional morphologies of the m‐SLE were characterized by SEM (MERLIN Compact, ZEISS, Germany) with EDS function. Optical images were acquired by an optical microscope (GX71, OLYMPUS). To evaluate the stretchability, a universal tensile tester (FT2000, Shanghai Mifang Electronic Technology Co., Ltd., China) was utilized, and electrical resistance was concurrently measured using a multimeter (Keysight 34420A). The initial volume conductivity was characterized with a surface resistivity meter (MCP‐T370 with ASP probe, Mitsubishi Chemical Corporation, Japan).

### Fabrication of the Finger Joint Strain Sensor

In order to fabricate a skin‐adhered strain sensor device, PDMS‐Ag was used to prepare strain‐sensitive structures for sensing and m‐SLE was used to prepare strain‐insensitive structures for interconnection. First, the complete pattern required for the sensor was EHD‐printed using Ag ink. After sintering the entire pattern, it was transferred to the surface of a PDMS substrate. Subsequently, the strain‐insensitive structures for electrodes were immersed in a Cu electrodeposition solution to obtain a bright Cu coating. EGaIn was applied to the surface of the Ag/Cu composite structure and selectively wetted the Cu surface. Copper foil tape was used to connect both ends of the interconnections to copper wires. To measure the resistance changes during two types of motions (Motion A and Motion B represent the bending of the first joint and the second joint respectively, as shown Figure 5d,e), copper wires were connected to a digital multimeter, and a series of resistance changes during the motions were recorded. The experiment obtained consent from all participants.

### Fabrication of the NFC Antenna

The NFC antenna for wireless energy harvesting was manufactured on a PDMS substrate (with PET support). Initially, the pattern of the NFC antenna was designed, and this circuit layer was subsequently fabricated using the m‐SLE preparation process. Then, an NFC chip (NTAG 215) and an indicative LED array were installed, displaying “HIT.” Finally, the NFC antenna was encapsulated with a PDMS film.

### Fabrication of the Stretchable Multilayer Display

A multi‐layer stretchable display with LEDs was manufactured. Initially, a circuit for a 3 × 5 LED array was designed, and this circuit layer was subsequently fabricated using the m‐SLE preparation process. Then, 15 surface‐mounted LEDs (Red, RISYM) were placed to the corresponding positions on the m‐SLE circuit. Finally, the circuit was encapsulated by spin‐coating a layer of PDMS solution on top. The integrated multi‐layer stretchable optoelectronic electronic skin display was simplified to a 3 × 5 LED array, controlled by an STM32F103C8T6 microcontroller. Various programs were available to control the triggered LEDs, displaying different letters or numbers.

## Conflict of Interest

The authors declare no conflict of interest.

## Supporting information

Supporting Information

Supplemental Video 1

Supplemental Video 2

Supplemental Video 3

Supplemental Video 4

Supplemental Video 5

## Data Availability

The data that support the findings of this study are available in the supplementary material of this article.
