# Supplementary material for: Microconfined Assembly of High‐Resolution and Mechanically Robust EGaIn Liquid Metal Stretchable Electrodes for Wearable Electronic Systems
Source: Adv Sci (Weinh). 2024 Jun 19;11(34):2402818. doi: 10.1002/advs.202402818 (PMC11425843; doi:10.1002/advs.202402818)
Supplement: Supplementary file 1 — Supporting Information [file ADVS-11-2402818-s003.pdf]

ADVANCED SCIENCE
Open Access

Supporting Information

for *Adv. Sci.*, DOI 10.1002/advs.202402818

Microconfined Assembly of High-Resolution and Mechanically Robust EGaIn Liquid Metal Stretchable Electrodes for Wearable Electronic Systems

Jingxuan Ma, Zicheng Sa, He Zhang, Jiayun Feng, Jiayue Wen, Shang Wang and Yanhong Tian**

Supporting Information

Microconfined assembly of high-resolution and mechanically robust EGaIn liquid metal stretchable electrodes for wearable electronic systems

Jingxuan Ma[1], Zicheng Sa[1], He Zhang[2,3], Jiayun Feng[1*], Jiayue Wen[4], Shang Wang[1,4], Yanhong Tian[1,4*]

1 National Key Laboratory of Precision Welding & Joining of Materials and Structures, Harbin Institute of Technology, Harbin, China, 150001

2 Department of Mechanical Engineering, The University of Hong Kong, Hong Kong SAR, China, 999077

3 Advanced Biomedical Instrumentation Centre Limited, Hong Kong SAR, China, 999077

4 Zhengzhou Research Institute, Harbin Institute of Technology, Zhengzhou, China, 450041

**Email: fengjy@hit.edu.cn; tianyh@hit.edu.cn, Tel: +86-451-86418359*

Table of Contents

Supplementary Figures

Figure S1. Material characterization of AgNPs.

Figure S2. EHD printing process of Ag-based conductive ink.

Figure S3. Current density optimization of Cu electrodeposition.

Figure S4. Selective wetting process of liquid metal on Cu surface.

Figure S5. Representative m-SLE patterns.

Figure S6. Tunable printed line width and pitch.

Figure S7. SEM and surface scanning images of m-SLEs prepared from different types of liquid metals and elastomers.

Figure S8. Optical microscope images of different stages of m-SLE fabrication process.

Figure S9. The cross-sectional EDS mapping images of m-SLE.

Figure S10. PDMS-Ag and m-SLE tensile test samples.

Figure S11. One stretching cycle of the m-SLE sample (10,000 cycles in total).

Figure S12. Cycling stability of PDMS-Ag sensor at 30% strain within 10000 cycles. The insets show the first and last 20 cycles respectively.

Figure S13. Long term stability of PDMS-Ag and m-SLE.

Figure S14. Optical image showing the skin sensor device. The red and blue regions indicate the interconnection (PDMS-Ag) and strain sensor (m-SLE), respectively.

Figure S15. Optical photos of m-SLE based NFC antenna.

Figure S16. Flexible m-SLE based NFC antenna.

Supplementary Table

Table S1. Comparison of the resolution, number of functional layers, and cycling stability with reported literature.

Supplementary Videos

Video S1. EHD printing process of Ag pattern.

Video S2. Selective wetting process of liquid metal on Cu surface.

Video S3. The finger joint strain sensor using strain-sensitive elements and strain-insensitive interconnections.

Video S4. Wireless energy harvesting through NFC antenna.

Video S5. Highly integrated multilayer stretchable optoelectric E-skin.

Supplementary Figures

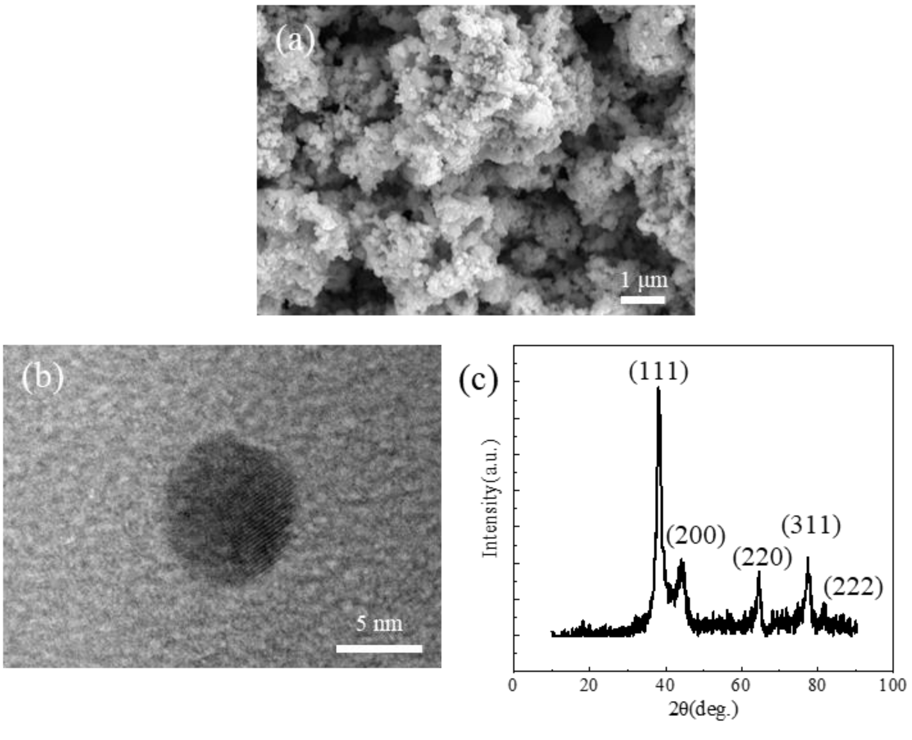

Figure S1. Material characterization of AgNPs. a) SEM image of AgNPs. b) High resolution TEM image of AgNPs. c) Powder XRD spectra of AgNPs.

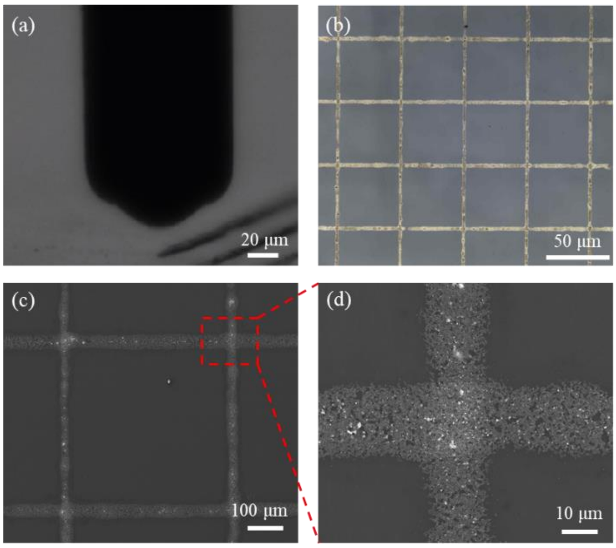

Figure S2. EHD printing process of Ag-based conductive ink. a) Stable Taylor cone during printing. b) Optical microscope image of Ag grid patterns. c) SEM image of Ag grid patterns. d) Enlarged SEM image of Ag grid patterns.

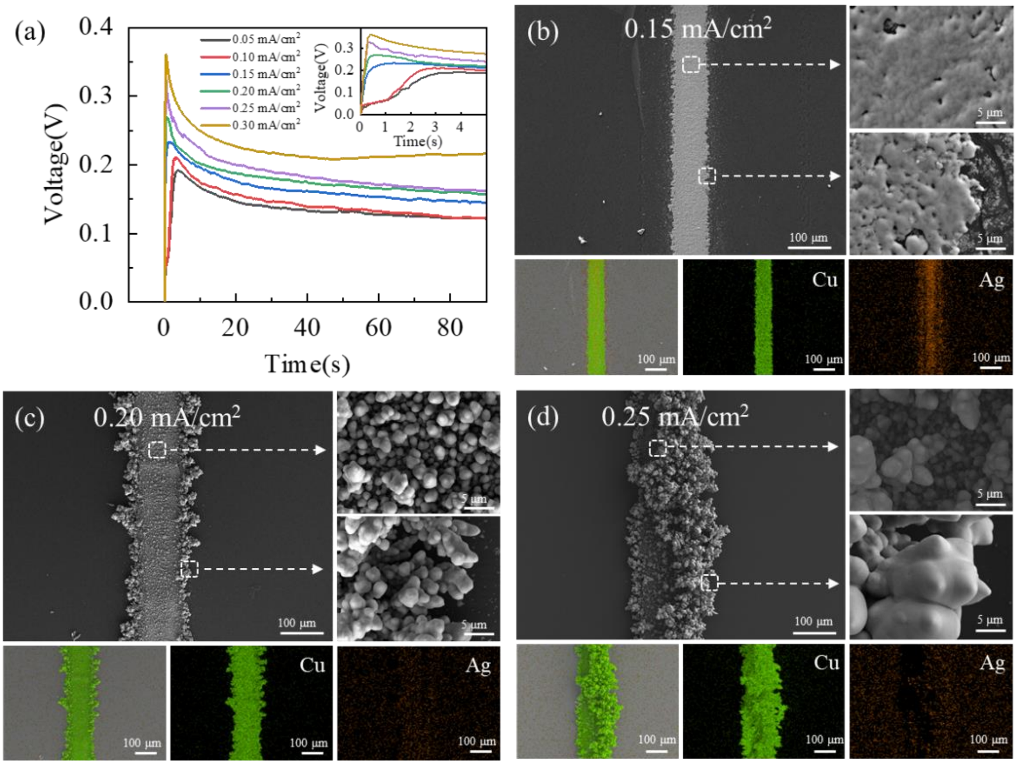

Figure S3. Current density optimization of Cu electrodeposition. a) Voltage changes during Cu electrodeposition at different current density (0.05, 0.10, 0.15, 0.20, 0.25, 0.30 mA/cm^2). b) SEM and surface scanning results under 0.15 mA/cm^2. c) SEM and

surface scanning results under 0.20 mA/cm^2. d) SEM and surface scanning results under 0.25 mA/cm^2.

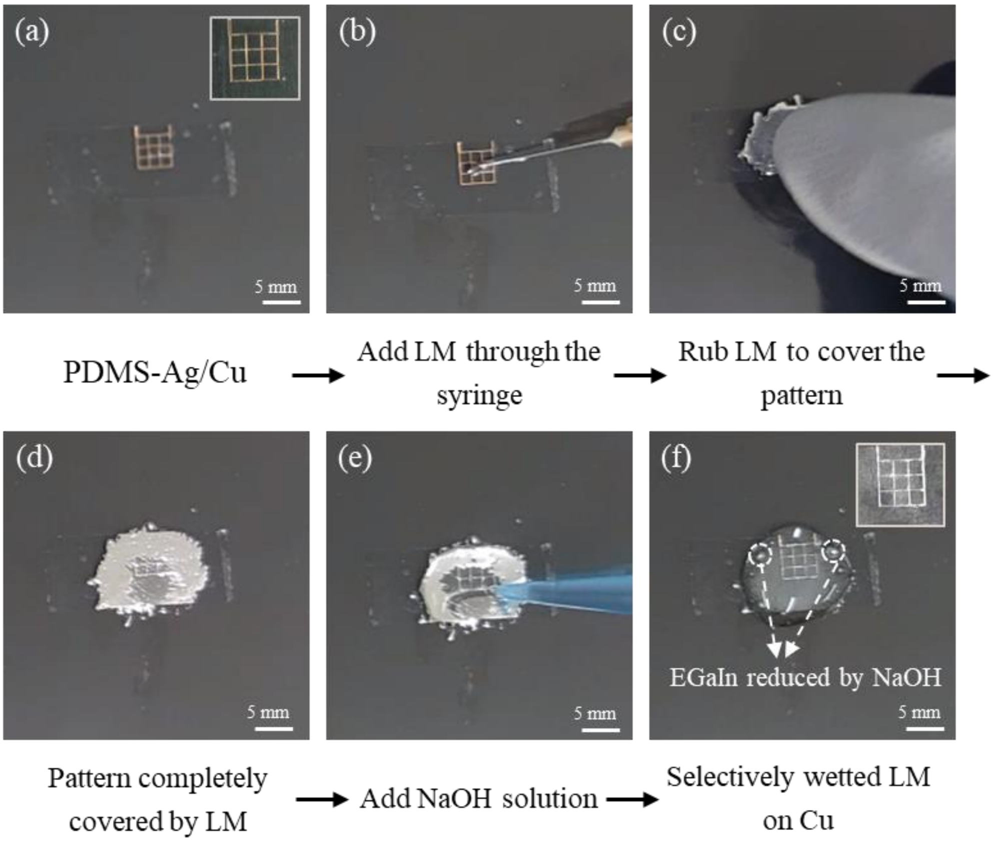

Figure S4. Selective wetting process of liquid metal on Cu surface. a) Initial PDMS-Ag/Cu pattern. b) The adding of LM through the syringe. c) Rub LM to cover the pattern. d) Pattern completely covered by LM. e) Add NaOH solution. f) Selectively wetted LM on Cu.

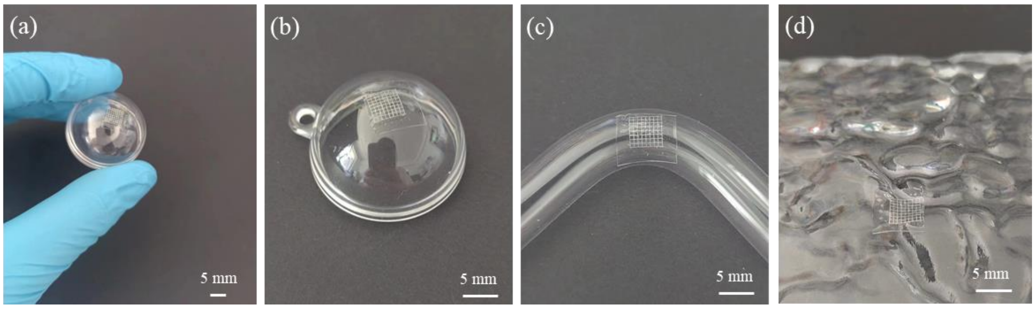

Figure S5. Representative m-SLE patterns. a) m-SLE structures on a glass hemisphere. b) Enlarged image. c) m-SLE structures on the corner of a glass bent

tube. d) m-SLE structures on a corrugated surface.

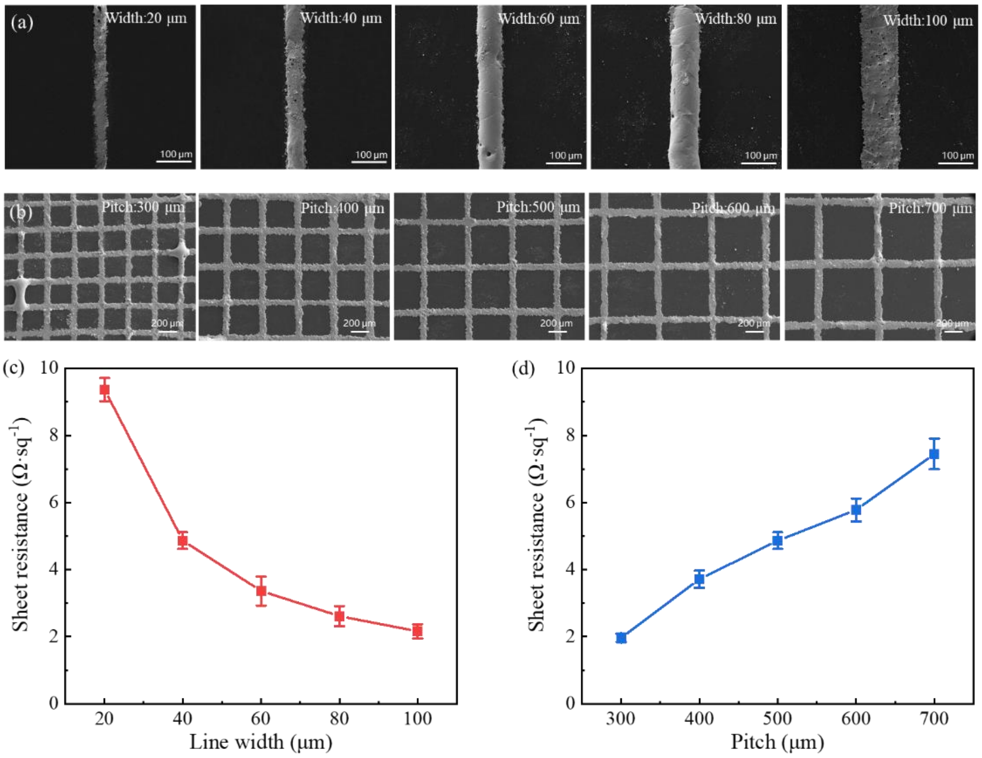

Figure S6. Tunable printed line width and pitch. a) Different printed sizes with line widths of 20, 40, 60, 80, 100 μm. b) Different print densities with pitches of 300, 400, 500, 600, 700 μm. c) Variations in sheet resistance with the line width. d) Variations in sheet resistance with the pitch.

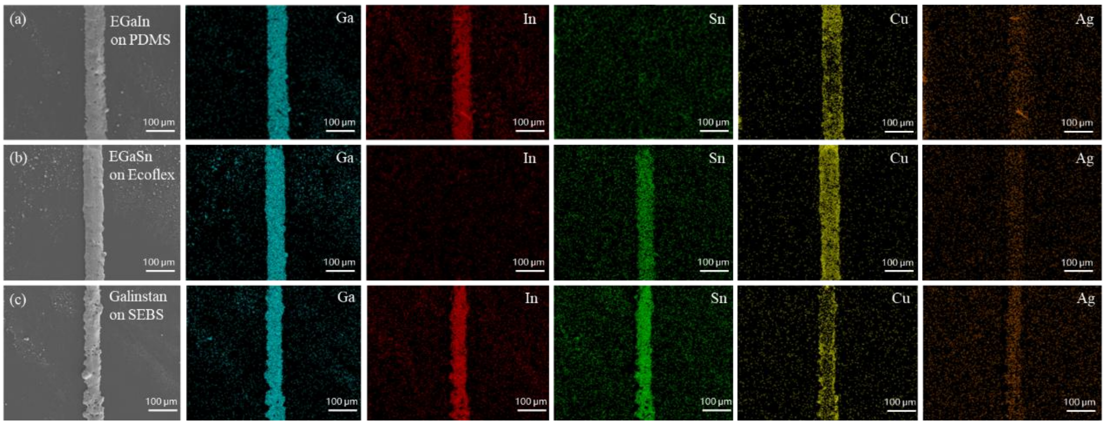

Figure S7. SEM and surface scanning images of m-SLEs prepared from different types of liquid metals and elastomers. a) EGaIn on PDMS. b) EGaSn on Ecoflex. c) Galinstan on SEBS.

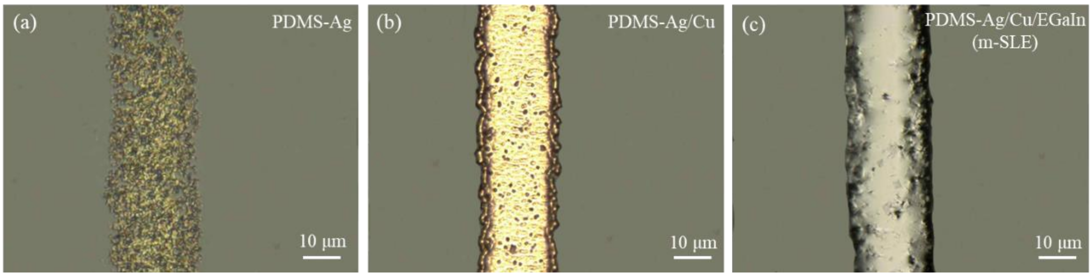

Figure S8. Optical microscope images of different stages of m-SLE fabrication process. a) PDMS-Ag. b) PDMS-Ag/Cu. c) PDMS-Ag/Cu/EGaIn (m-SLE).

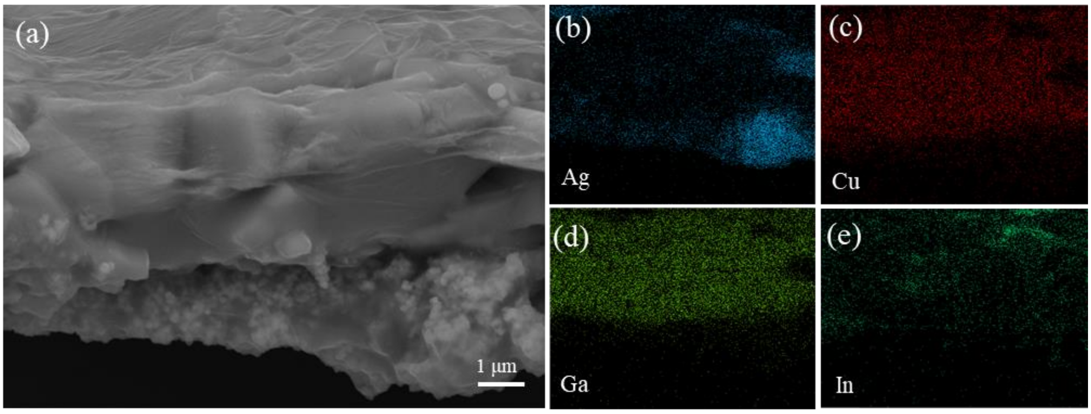

Figure S9. The cross-sectional EDS mapping images of m-SLE. a) Cross-sectional SEM image of m-SLE. b-d) Ag, Cu, Ga, In EDS mapping images.

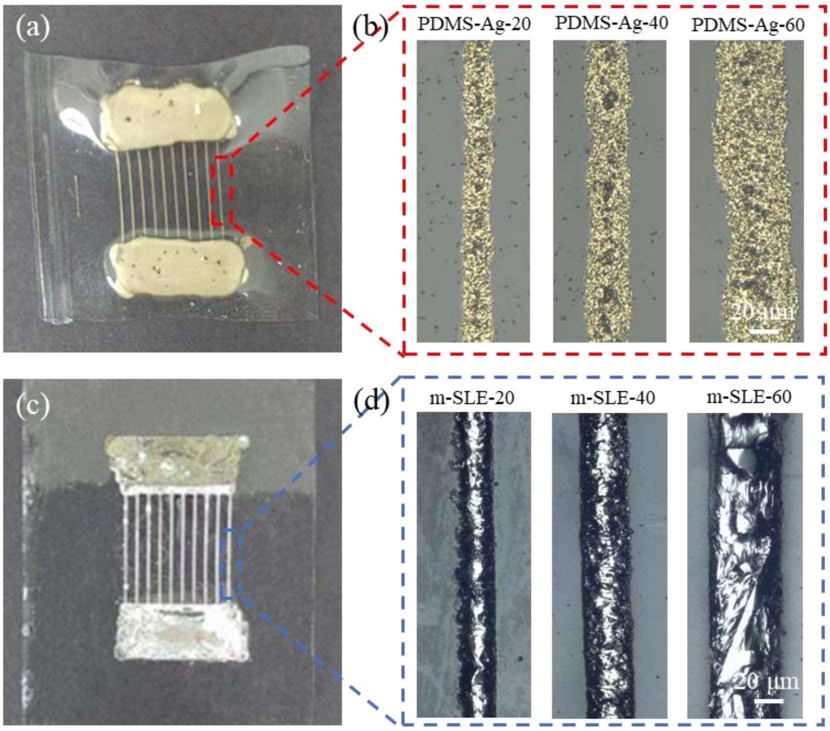

Figure S10. PDMS-Ag and m-SLE tensile test samples. a) Photos of PDMS-Ag tensile

test sample. b) Optical microscope images of PDMS-Ag with different linewidths. The samples with linewidths of 20, 40 and 60 μm are named PDMS-Ag-20, PDMS-Ag-40 and PDMS-Ag-60 respectively. c) Photos of m-SLE tensile test sample. d) Optical microscope images of m-SLE with different linewidths. The samples with linewidths of 20, 40 and 60 μm are named m-SLE-20, m-SLE-40 and m-SLE-60 respectively.

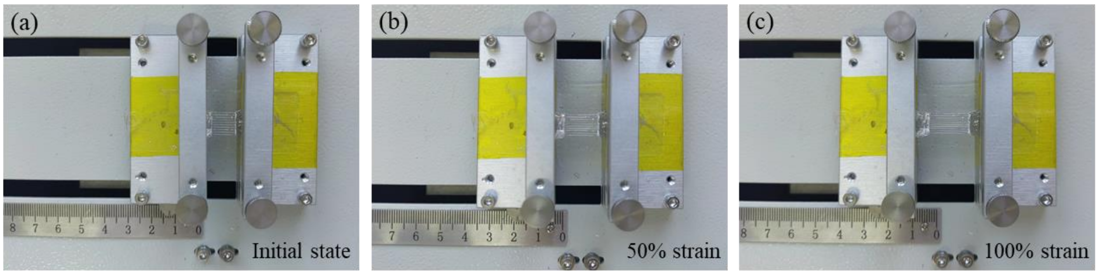

Figure S11. One stretching cycle of the m-SLE sample (10,000 cycles in total). a) Initial state. b) 50% tensile strain. c) 100% tensile strain.

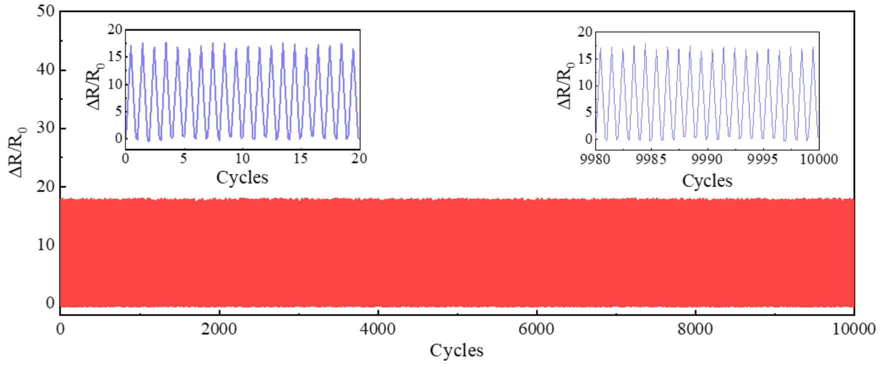

Figure S12. Cycling stability of PDMS-Ag sensor at 30% strain within 10000 cycles. The insets show the first and last 20 cycles respectively.

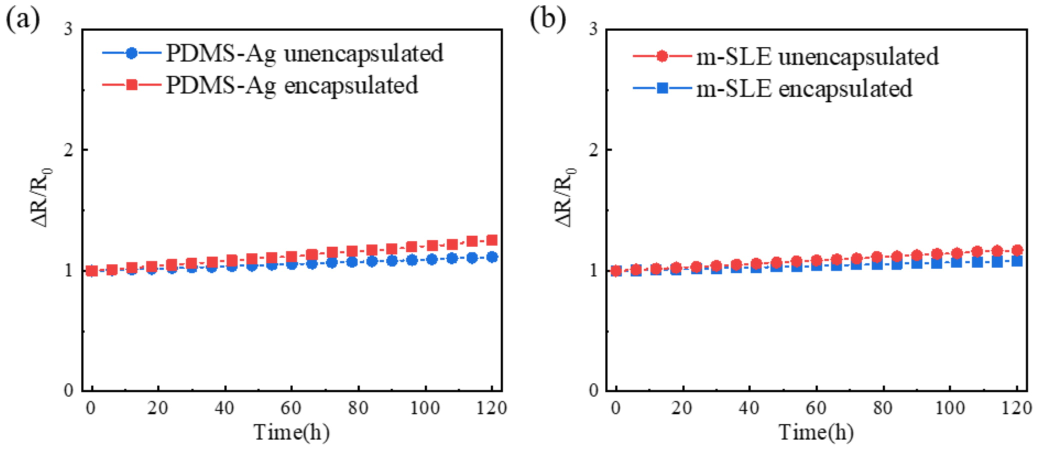

Figure S13. Long term stability of PDMS-Ag and m-SLE. a) Relative resistance variation of PDMS-Ag after storage in atmospheric environment. b) Relative resistance

variation of m-SLE after storage in atmospheric environment.

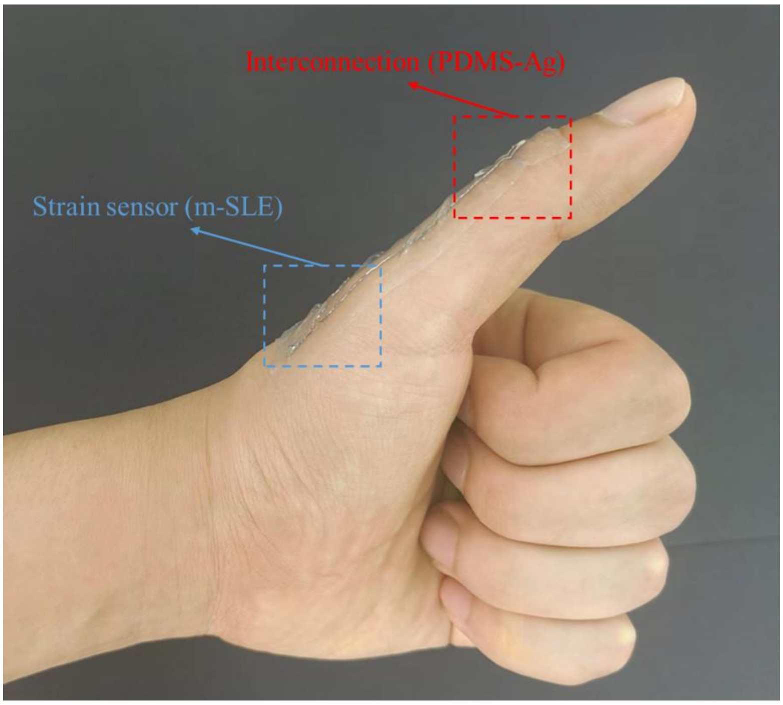

Figure S14. Optical image showing the skin sensor device. The red and blue regions indicate the interconnection (PDMS-Ag) and strain sensor (m-SLE), respectively.

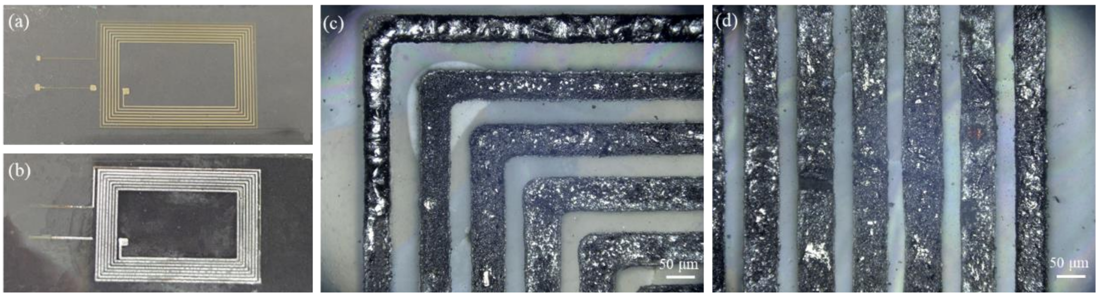

Figure S15. Optical photos of m-SLE based NFC antenna. a) PDMS-Ag based NFC antenna (without Cu and EGaIn treatment). b) m-SLE based NFC antenna. c) Optical microscope images of corners. d) Optical microscope images of straight lines.

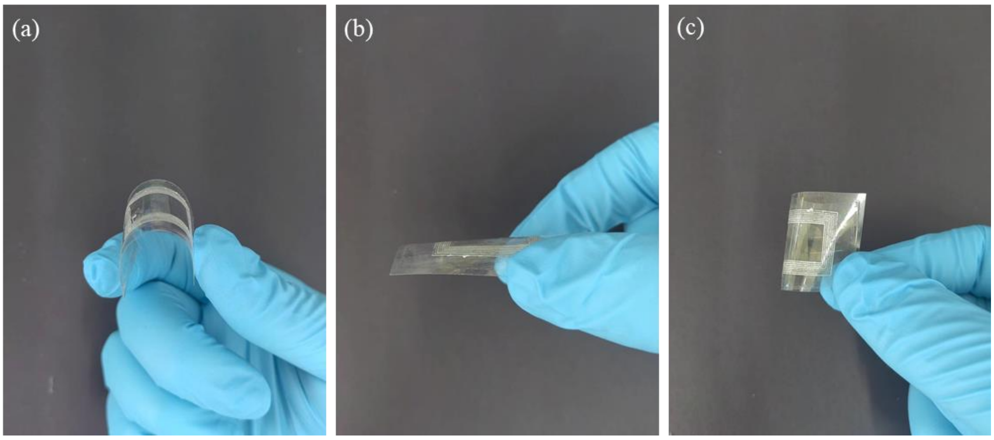

Figure S16. Flexible m-SLE based NFC antenna. a-b) NFC antenna bent at large angles. c) NFC antenna twisted at large angles.

Supplementary Table

Table S1. Comparison of the resolution, number of functional layers, and cycling stability with reported literature.

Resolution/μm	Layers	Cycles	Patterning method	Ref.
19.28	4	10000	EHD printing	This work
~1000	2	20	3D molding	53
~500	3	300	Stencil printing	54
200	2	10000	Stencil printing	55
~500	3	1010	Stencil printing	56
80	1	1000	Stencil printing	57
~600	2	200	Inkjet printing	58
~400	2	2000	Mask printing	59
~1000	2	1000	Direct writing	60
~100	3	10000	Direct writing	61
50	2	7500	Direct writing	62
300	2	1000	Direct writing	63
1.3	3	40	Photolithography	64
20	2	2000	Photolithography	65

Supplementary Videos

Video S1. EHD printing process of Ag pattern.

Video S2. Selective wetting process of liquid metal on Cu surface.

Video S3. The finger joint strain sensor using strain-sensitive elements and strain-insensitive interconnections.

Video S4. Wireless energy harvesting through NFC antenna.

Video S5. Highly integrated multilayer stretchable optoelectric E-skin.
